# Lactadherin Inhibits Secretory Phospholipase A_2_ Activity on Pre-Apoptotic Leukemia Cells

**DOI:** 10.1371/journal.pone.0077143

**Published:** 2013-10-23

**Authors:** Steffen Nyegaard, Valerie A. Novakovic, Jan T. Rasmussen, Gary E. Gilbert

**Affiliations:** 1 Department of Molecular Biology, Aarhus University, Aarhus C, Denmark; 2 Departments of Medicine, Veterans Administration Boston Healthcare System, Brigham and Women’s Hospital and Harvard Medical School, Boston, Massachusetts, United States of America; Universidade Federal do Rio de Janeiro, Brazil

## Abstract

Secretory phospholipase A_2_ (sPLA_2_) is a critical component of insect and snake venoms and is secreted by mammalian leukocytes during inflammation. Elevated secretory PLA_2_ concentrations are associated with autoimmune diseases and septic shock. Many sPLA_2_’s do not bind to plasma membranes of quiescent cells but bind and digest phospholipids on the membranes of stimulated or apoptotic cells. The capacity of these phospholipases to digest membranes of stimulated or apoptotic cells correlates to the exposure of phosphatidylserine. In the present study, the ability of the phosphatidyl-L-serine-binding protein, lactadherin to inhibit phospholipase enzyme activity has been assessed. Inhibition of human secretory phospholipase A_2_-V on phospholipid vesicles exceeded 90%, whereas inhibition of *Naja mossambica* sPLA_2_ plateaued at 50–60%. Lactadherin inhibited 45% of activity of *Naja mossambica* sPLA_2_ and >70% of human secretory phospholipase A_2_-V on the membranes of human NB4 leukemia cells treated with calcium ionophore A23187. The data indicate that lactadherin may decrease inflammation by inhibiting sPLA_2_.

## Introduction

Secretory phospholipase A_2_ (sPLA_2_) is a nine-member family of 16–18 kDa enzymes with five to eight disulfide bonds. These Ca^2+^-dependent enzymes hydrolyze *sn*-2 esters of phospholipid molecules via an Asp-His diad [1]–[3]. The action of sPLA_2_ occurs in two phases. First, the sPLA_2_ binds to a bilayer and then the bound molecule continues predominantly in scooting mode, cleaving multiple phospholipid substrate molecules without dissociating from the membrane [4]. A substantial portion of membrane and phospholipid specificity is exhibited in the initial binding step. Thus, one mechanism of inhibiting activity of secretory phospholipase A_2_ is via competition for the initial membrane binding sites.

Of the family of sPLA_2_’s, three are of particular interest in relation to inflammation, namely group IIA (hsPLA_2_-IIA), group V and group X. Group IIA (also known as synovial sPLA_2_ or non-pancreatic PLA_2_) is a secreted phospholipase originally found and purified from synovial fluid of rheumatoid arthritis patients and correlates with sepsis as well as autoimmune disease [5]–[10]. The basic group IIA sPLA_2_’s are highly selective for anionic phospholipids, with phosphatidylserine (PS) as an important phospholipid target for efficient plasma membrane docking [4], [11], [12]. Comparably, group V is closely related to group IIA [13], but more neutral and maintains efficient docking via a tryptophan in the docking interface [14], [15]. In a mechanistically similar fashion, group X exhibits more hydrophobic residues allowing docking to and hydrolysis of zwitterionic phospholipids like phosphatidylcholine and is largely unaffected by PS exposure [16]. Together, the native charge as well as interface residues largely account for the preferred substrate of these three enzymes, with group V and X more readily binding and cleaving zwitterionic substrates and generating the gateway molecule of eicosanoid synthesis, arachidonic acid, as compared to group IIA [17], [18].

Although all three isoforms are relevant in inflammation, group V sPLA_2_ is a good candidate as it depends on both PS dependent and hydrophobic docking mechanisms while also yielding substantial arachidonic acid on leukocyte cell membranes [14], [15], [19], [20]. As reviewed by Murakami and Lambeau, *in vivo* studies on group V sPLA_2_ have expanded on the understanding of the role of group V sPLA_2_ in inflammation [21], [22]. Recent studies have shown group V to act in a proinflammatory fashion as expected from the higher arachidonic acid release as compared to group IIA, with group V being upregulated in asthma and showing a proinflammatory dose-response relationship upon aerosol administration of sPLA_2_-V [23]. Group V knock-out mice have however revealed an anti-inflammatory effect in a disease and tissue specific manner with decreased phagocytosis of IgG-opsonized sheep red blood cells is impaired in group V −/− macrophages [24] and a protective effect in K/BxN autoantibody-induced inflammatory arthritis [10]. Taken together, these findings indicate that inflammation originating from excessive sPLA_2_ activity should be addressed in a disease and tissue specific manner to avoid adverse effects and that a systemic therapeutic seems unlikely [25]–[27].

The inducing effect of PS on group IIA and V activity is minimized in quiescent cells by aminophospholipid translocases which keep PS sequestered on the inner leaflet of the plasma membranes of quiescent cells making it inaccessible to secretory phospholipase A_2_ [28]–[31]. PS-exposure on the outer leaflet of the plasma membrane is observed starting in early apoptosis as a response to cellular stress [32], [33]. Accordingly, it has been demonstrated that PS-exposure influences the production of arachidonic acid and thereby synthesis of proinflammatory downstream products like leukotrienes and prostaglandins [34]–[37]. These cyto- and chemokines are an important part of the inflammatory cascade leading to increased vascular permeability, recruitment of leukocytes, modulation of clotting, and induced mast cell chemokine production [38]. Thus, inflammatory function of secretory phospholipase A_2_ is linked to cell stimulation and apoptosis via PS exposure.

Studies of the relationship of PS exposure to activity of PLA_2_’s can be aided by reagents that report PS exposure as well as those that block PS, preventing access of PLA_2_. Accordingly, it has been shown that lactadherin (also called MFG-E8) is a sensitive and selective probe for PS, with the bovine ortholog being the most extensively studied [39]–[42]. Bovine lactadherin is a 409 amino acid protein that can be purified as two glycosylation variants (47 and 52 kDa) from bovine milk. This peripheral bound membrane protein is composed of two N-terminal EGF-like domains, with an integrin binding RGD sequence in the EGF-2 domain [40], [43]. Tandem discoidin-like domains, C1 and C2, mediate membrane binding. Lactadherin exhibits a strong affinity for PS-containing membranes with a K_d_∼0.08–4 nM [42], [43]. Lactadherin also displays stereospecific binding to phosphatidyl-L-serine and preference for convex membranes. Membrane binding of lactadherin is not Ca^2+^-dependent which further improves its value for monitoring and blocking exofacial PS [41], [44]–[46]. There are structural and functional similarities between the C1 and C2 domains of lactadherin and those of blood coagulation factors V and VIII [39], [41], [42]. The structural homology of lactadherin with factor V and factor VIII correlates with the capacity of lactadherin to compete for PS-containing membrane binding sites and to function as a potent anticoagulant [45]. The integrin and PS binding enables lactadherin to operate as an opsonin, by bridging apoptotic cells and vesicular debris, with exposed PS, to phagocytic immune cells [47], [48].

The research presented in this study aims at investigating the extent to which lactadherin affects the activity of sPLA_2_. Accordingly, activity was studied on vesicles of varying composition and size as well as on immortalized, human NB4 leukemia cells. The results indicate that lactadherin inhibits the enzymatic activity of phospholipase A_2_-V and, to a lesser extent, a snake venom phospholipase A_2_.

## Materials and Methods

### Materials

Phosphatidylcholine (PC, egg yolk), phosphatidylethanolamine (PE, egg yolk) and phosphatidyl-L-serine (PS porcine brain) were purchased from Avanti Polar Lipids (AL, USA). 1,2-bis-(4,4-difluoro-5,7-dimethyl-4-bora-3a,4a-diaza-s-indacene-3-unde-canoyl)-sn-glycero-3-phosphocholine (bis-BODIPY FL C11-PC) was from Life technologies (NY, USA). Fatty acid free bovine serum albumin (BSA) was from EMD biosciences (Germany). Human factor Xa and prothrombin were purchased from Enzyme Research Laboratories (IN, USA), factor Va was purchased from Haematologic Technologies Inc. (VT, USA), and S-2238 thrombin chromogenic substrate from Diapharma (OH, USA). Calcium ionophore A23187, propidium iodide (PI) and *Naja mossambica* venom secretory phospholipase A_2_ (nmPLA_2_) were purchased from Sigma-Aldrich (MO, USA). Recombinant human secretory phospholipase A_2_ group V (hsPLA_2_-V) was purchased from Cayman chemical (MI, USA). Bovine lactadherin was purified and labeled with fluorescein isothiocyanate (FITC) as described previously [39], [49]. Human promyelocytic leukemia NB4 cells [50] were a generous gift from Dr J. O’Kelly (Los Angeles, CA). Acrylodan-labeled Intestinal Fatty Acid Binding Protein (ADIFAB) was purchased from FFA Sciences, (CA, USA). All other chemicals (analytical grade) were supplied by Sigma-Aldrich Corp. (St. Louis, Mo) or Merck and Co. Inc (Whitehouse Station, NJ).

### Lactadherin Purification

Lipid-free lactadherin was purified from fresh bovine milk essentially as described previously [39]. Purity was checked by SDS-PAGE showing the presence of only two bands at M_r_ 47 and 52 kDa corresponding to the two glycosylation variants, and by N-terminal amino acid sequencing demonstrating more than 97 percent purity. Lactadherin concentration was determined by measuring at A_280 nm_ (ε = 77180 M^−1 ^cm^−1^, calculated) and stored at −80°C in 75 mM sodium phosphate, pH 7.0, 40 mM KCl.

### Phospholipid Vesicles

Sonicated small unilamellar and extruded large unilamellar phospholipid vesicles (PLVs) of composition PS:PE:PC:bis-BODIPY PC 4∶20:75∶1 were prepared as previously described [41]. Phospholipid concentration was determined by elemental phosphorous assay [51]. Vesicles were flash frozen in liquid nitrogen, stored at −80°C, and thawed at 37°C within 2 hr of experiments.

### Spectrofluoroscopic Assay of Secretory PLA_2_ Activity on Vesicles

The ability of lactadherin to inhibit sPLA_2_’s was determined using various concentrations of purified lactadherin against nmPLA_2_ and hsPLA_2_-V. Recombinant human phospholipase A_2_ group V was chosen due to the apparent involvement of hsPLA_2_–V in eicosanoid synthesis. Phospholipase activity was measured on a Peltier-thermostatted QMC-4-CW spectrofluorometer (PTI, NJ, USA) with λex 488 nm and λem 515 nm. Slit widths were 0.5 mm for excitation and 1 mm for emission. Unless otherwise indicated, phospholipid vesicles concentrations were 10 µM, phospholipase concentration 0.06 U/ml, with enzyme kept on ice and experiments done in duplicates. Measuring buffer was HEPES (20 mM HEPES, 140 mM NaCl, 5 mM KCl, 1 mM Na_2_HPO_4_, 1.5 mM CaCl_2_ and MgCl_2_–pH 7.4) for vesicle experiments.

PLVs were allowed to equilibrate at 4°C in 150 µl measuring buffer for 5 min in the dark prior to addition of sPLA_2_ and lactadherin in 150 µl, pre-cooled to 2°C. Vesicles were added via an injection port so that fluorescence monitoring was not interrupted. Fluorescence intensity dropped with injection, in proportion to dilution of the PLV. Fluorescence intensity was recorded in kinetic mode and analyzed for 6 min. at 4°C immediately following the dilution-related decrease in intensity. Fluorescence data were normalized to the value immediately following PLA_2_ injection for display and analysis. When adding lactadherin, the maximum activity of the inhibited reaction is expressed against the uninhibited maximum activity.

### Cell Culture

NB4 cells were maintained in RPMI 1640, 10% v/v fetal bovine serum, 1% v/v 5,000 U/ml 5,000 µg/ml penicillin and streptomycin (Life technologies, NY, USA) at 37°C, 5% CO_2_, humidified atmosphere in a Symphony incubator (VWR, PA, USA). Optimal growth rate and cell health was ensured by sub-culturing 24 hours prior to experiments. All experiments were carried out between passage 17 and 25. Washes were done in no phenol red no serum RPMI1640 and centrifugation at 68 RCF with slow acceleration and deceleration to avoid premature stress. Cells were treated with calcium ionophore A23187 which in turn causes PS exposure on the cytofacial surface [52], [53]. This procedure has previously been used successfully to mimic the PS exposure of apoptotic cells in phospholipase assays [20], [54], [55] and produce a stressed phenotype comparable to stimulated platelets [52]. A23187 was used at 6 µM concentration and added to the cell suspension 10 minutes prior to experiments. Optimal ionophore concentration and incubation time were titrated by prothrombinase assay and FITC-labeled lactadherin as described below. An alternate cell stress protocol was employed to discount interactions between A23187 and lactadherin. NB4 cells were stressed by incubation for 6 hours in RPMI 1640 containing 40 µM etoposide before being washed gently twice in 37°C no phenol red no serum RPMI 1640 as previously described. Five times the original volume of cell suspension was used (incubated cell suspension was 2 ml and wash carried out using 2×10 ml RPMI 1640) insuring that little etoposide remained. The 40 µM etoposide concentration was loosely based on Kaufmann et al. [56] and concentration and incubation time verified by PI/FITC-lactadherin flow cytometry as previously described.

### Prothrombinase Assay of Cellular PS Exposure

PS exposure was measured using a two-step amidolytic substrate assay for prothrombinase activity where the exposed membrane PS is the limiting component of the prothrombinase complex [57]. The NB4 cells were gently washed, resuspended into reaction buffer (No serum no phenol red RPMI1640, 0.005% v/v BSA) and 100,000 cells added per well. A mixture of 0.5 nM Factor Xa and 0.5 nM Factor Va in reaction buffer was added to each well followed by substrate mix of 1 µM prothrombin and 1.5 mM Ca^2+^. The reaction was incubated for 5 min at room temperature and terminated by adding stop buffer (20 mM Tris/HCl - pH 7.0, 0.005% BSA and 16 mM EDTA) to each well. The chromogenic substrate S-2238 was added in each well to 115 µM and the speed of color generation was measured at 405 nm using a VersaMAX microplate reader (Molecular Devices, CA, USA) in kinetic mode, 3 second intervals. The reaction rate was found using Softmax Pro and an appropriate timeframe (R^2^>0.9).

### Flow Cytometry Assay of Cellular PS Exposure

PI is traditionally used as an apoptosis marker [58], however as the distinction between late stage apoptosis and necrosis can be difficult, the combination of lactadherin and PI was used to allow full control over the cell death pathway. Cell stress quantified by PS exposure was monitored over time using FITC labeled Lactadherin. Labeling was done as described previously [44], [49] with FITC-lactadherin and PI used as apoptosis marker in a similar fashion as Vermes et al, Fadok et al a.o. [59]–[61]. 2×10^6^/ml NB4 cells were washed gently in RPMI 1640 without serum or phenol red and immediately treated using 6 µM A23187. Treated cells were monitored for 90 minutes on a Becton Dickinson LSRFortessa flow cytometer (BD biosciences, CA, USA) using 10 nM FITC-lactadherin and 2 µg/ml PI. Data processing was done using FACS Diva and FCS Express.

### Spectrofluoroscopic Assay of Secretory PLA_2_ Activity on NB4 Cells

The rate of free fatty acid release by NB4 cells was monitored by the fluorescence change of ADIFAB. Reactions were analyzed with 100,000 cells per/ml in a 3∶1 mix of HEPES-buffered saline and RPMI1640 adjusted to 1.15 mM CaCl_2_. The reaction volume was 300 µl in 3×3 mm micro square cell cuvette at 37°C. Fluorometer settings were λex 380 nm and λem 440 nm with slit widths of 0.5 mm and 1.3 mm for excitation and emission, respectively. Activity was initiated by injecting 250 µl sPLA2 in 50 µl cell solution over approx. 3 s. The final sPLA2 concentration was 0.06 U/ml. Due to the very fast initial reaction rate of the stressed NB4 cells, the maximum activity was determined by obtaining the slope by linear regression and extrapolating back the last measured value prior to injection, usually 3–4 seconds.

### Dataprocessing and Statistical Analysis

Standardized curves were fitted to a double exponential association model:
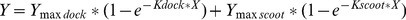
(1)


Normalized curves (inhibition curves) were fitted to a double exponential association model:
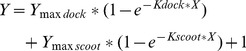
(2)


Initial rates versus substrate concentration was fitted to a Michalis-Menten equation:
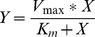
(3)


As the reaction was very rapid on live cell membranes the initial part of the curves were missing. The initial starting point of the reaction curves was found by linear extrapolation back to the original injection point. This was done to acquire the correct maximal RFU value (ΔY) using:
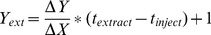
(4)


All error bars display standard deviation and significance was calculated using Welch’s t-test. Data processing was done in Microsoft Excel 2010 and Graphpad Prism 5.

## Results

**Figure 1:**
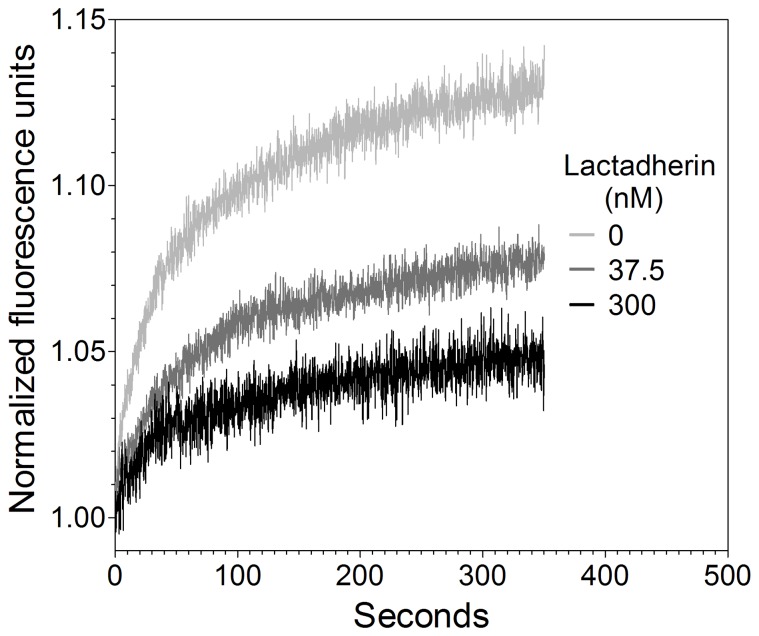
Cleavage of fluorescent phospholipid by nmPLA_2_ and inhibition by lactadherin. Sonicated phospholipid vesicles, 10 µM, of composition PS:PE:PC:bbPC 4∶20:75∶1 were pre-incubated for 15 minutes in PBS, pH 7.2 at 22°C with, or without, pure lipid-free bovine lactadherin before addition to a quartz cuvette of 3×3×45 mm. Vesicles were incubated for 5 minutes at 4°C in the Peltier-thermostatted sample chamber of the fluorometer before adding nmPLA_2_ to a final concentration of 0.06 U/ml. Reaction curves are normalized to baseline fluorescence intensity before addition of phospholipase as per Eq. 2.

**Figure 2:**
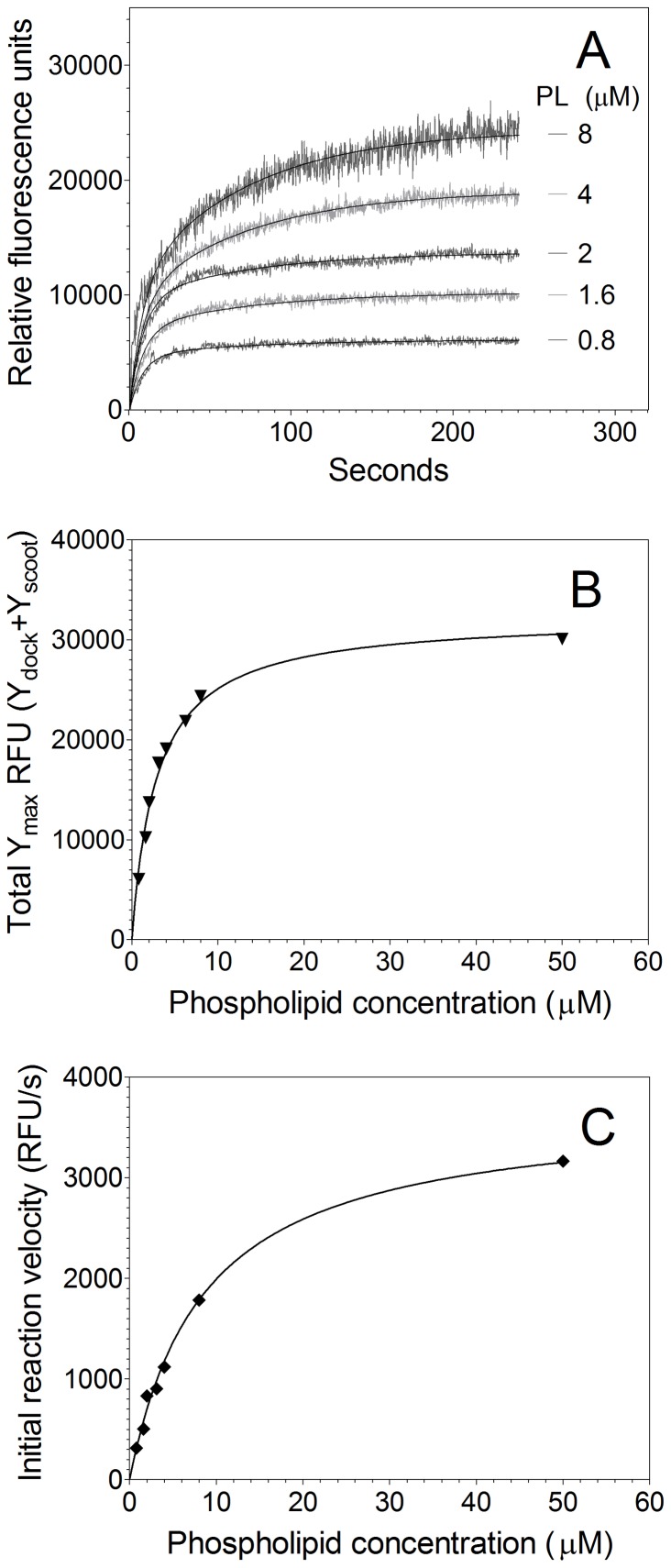
Relationship between PLA_2_ concentration, phospholipid concentration, and phospholipase activity. (**A**) Varying concentrations of sonicated vesicles of composition PS:PE:PC:bbPC 4∶20:75∶1 were allowed to equilibrate at 4°C for 5 minutes before adding 0.125 U/ml nmPLA_2_. The resulting curve replicates were standardized and fitted to a two-phase exponential association model (Eq. 1) and solved for global rate constants (fitted curves). (**B**) Total Y_max_ obtained from fitted curves was plotted against phospholipid concentrations and fitted to a Michaelis-Menten equation. (**C**) The initial reaction rate was obtained from the original datasets using linear regression from 0–5 seconds and plotted against phospholipid concentration. A Michaelis-Menten equation was fitted to the data and of high fit quality. The results indicate a saturable dose-response relationship. Experiments at each phospholipid or phospholipase concentration were performed a minimum of two times, and values averaged.

**Figure 3:**
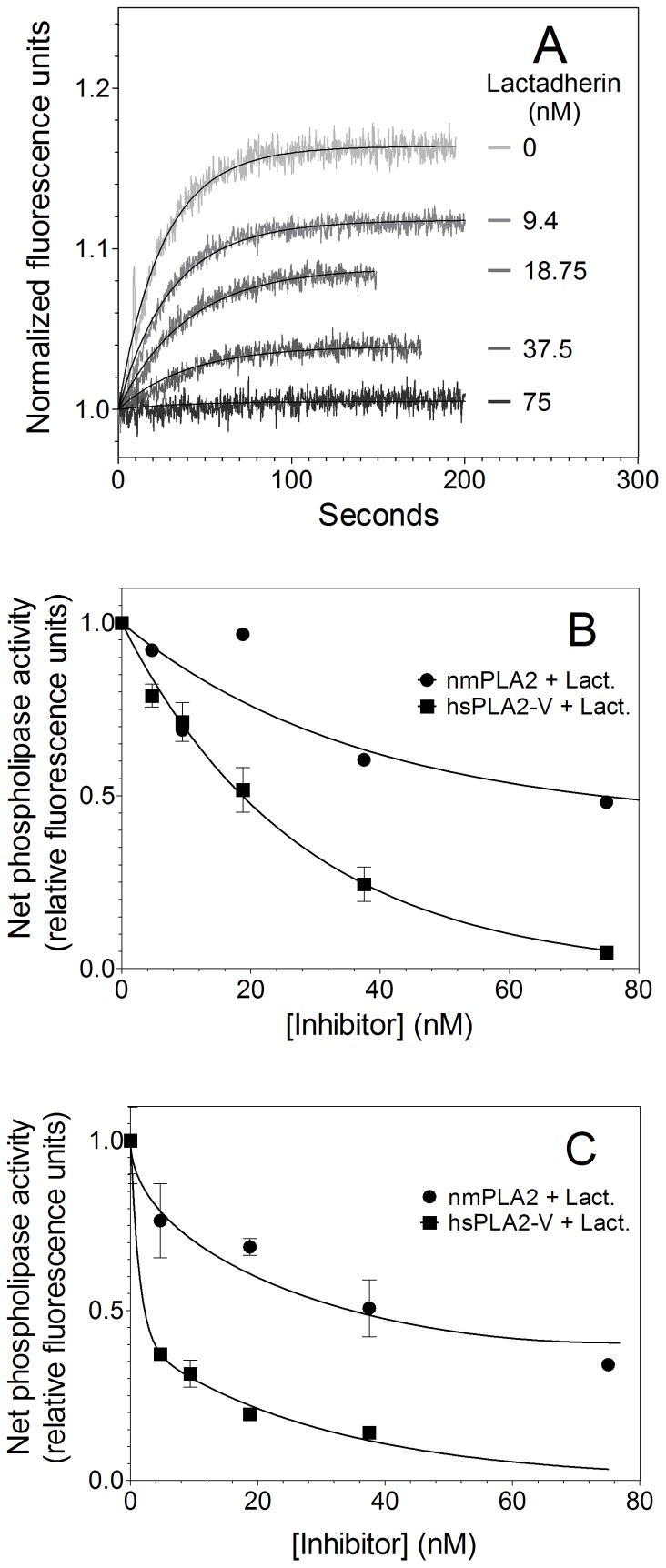
Lactadherin inhibition of hsPLA_2_ vs. nmPLA_2_. (**A**) Various concentrations of lactadherin were added to 10 µM phospholipid vesicles with composition as described for Fig. 2. Phospholipid vesicles were preincubated for 15 minutes with bovine lactadherin before transfer to a quartz cuvette and cooling for 5 minutes at 4°C. hsPLA_2_-V was added to a final concentration of 0.06 U/ml and the activity monitored continuously as fluorescence emission at 515 nm. Data were fitted to a double exponential model and fitted globally as described in Fig. 2. Addition of lactadherin diminished both components of enzyme activity. (**B**) Normalized net phospholipase activity on sonicated PLVs after 400 s ± SD are plotted as a function of lactadherin concentration with uninhibited Y_max total_ as reference. (**C**) Experiments like those in panel 3A were performed utilizing extruded PLV rather than sonicated vesicles (not shown). Normalized phospholipase activity after 400 s ± SD are plotted as function of lactadherin concentration. As seen in all panels, the PS sensitive hsPLA_2_-V is more readily blocked by lactadherin. All experiments were performed at least twice and results averaged for data displayed in panels B and C.

We wished to determine whether lactadherin has the capacity to inhibit activity of secretory PLA_2_. Accordingly, we developed a real-time fluorescence assay and tested activity of a secretory PLA_2_ from the venom of *Naja mozambiqua* (nmPLA_2_) toward phospholipid vesicles containing 4% PS (Fig. 1). This membrane composition is similar to the outer leaflet of apoptotic cells that have undergone stimulation or stress [33], [62]. Our results indicated that nmPLA_2_ rapidly cleaves a fraction of the fluorescent bis-BODIPY PC. At 37°C and at 25°C the fluorescence change approached completion within 5 s, making it difficult to record the details. Thus, the experimental data obtained for Figures 1–3 was obtained at 4°C, conditions under which substrate cleavage occurred over 6 minutes. Addition of lactadherin diminished the initial rate of substrate cleavage and the plateau. Thus, lactadherin has the capacity to inhibit activity of at least one secretory PLA_2_.

The relationship between nmPLA_2_ concentration and vesicle concentration was explored in order to determine optimal conditions for evaluating the inhibitory effect of lactadherin (Fig. 2). The data were normalized to initial fluorescence to emphasize the impact of varying phospholipid substrate with a fixed phospholipase concentration. Results indicated that the relative rate of substrate cleavage and the plateau are both influenced by the initial phospholipid concentration (Fig. 2A). Thus, initial phospholipase activity increased with substrate concentration then slowed primarily in response to factor(s) other than substrate depletion. This was explored by plotting the combined Y_max_ obtained from a two-phase exponential association fit (Eq. 1) versus phospholipid concentration (Fig. 2B). To verify that the initial kinetic component was obeying Michaelis-Menten kinetics under our experimental conditions, initial reaction rates from 0 s to 5 s were measured and plotted against phospholipid concentration (Fig. 2C) and fitted to a Michealis-Menten equation (Eq. 3), R^2^ = 0.96. Similar curves were obtained with hsPLA_2_-V (data not shown). Thus, under these conditions the number of sPLA_2_ molecules/membrane phospholipid influences the total substrate cleavage as well as the initial rate. These results indicate the relationship between the parameters of the assay and the rate of substrate cleavage, enabling more quantitative testing of the inhibition by lactadherin. Further experiments were designed to primarily affect the initial, rapid component of phospholipase activity.

We tested the inhibitory activity of lactadherin toward human secretory PLA_2_ (Fig. 3). The results indicated progressive inhibition at increasing concentrations of lactadherin with greater than 95% inhibition at 75 nM lactadherin on 4% PS SUV (Fig. 3A). A direct comparison of the inhibitory capacity of lactadherin toward nmPLA_2_ vs. hsPLA_2_-V confirmed that 75 nM lactadherin inhibits approx. 50% nmPLA_2_ activity while inhibiting greater than 95% hsPLA_2_-V activity on 4% PS SUV (Fig. 3B). Because the binding of lactadherin and PLA_2_’s are both sensitive to membrane curvature [41], [63] we asked whether inhibition by lactadherin differed on extruded vesicles, with lesser membrane convexity than sonicated vesicles (Fig. 3C). The relative fluorescence increase, in response to PLA_2_, with extruded vesicles was approx. 3-fold greater than with sonicated vesicles, possibly related to a greater degree of self-quenching by the BODIPY acyl chains in the more tightly packed membrane cores of extruded vesicles. Lactadherin inhibition studies indicated that lower concentrations inhibit hsPLA_2_-V on extruded vesicles with IC50<4 nM vs. 18 nM on sonicated vesicles. The IC50 for nmPLA_2_ was also lower, by approx. 2-fold on extruded vesicles. These results indicate that lactadherin inhibits hsPLA_2_-V more effectively than nmPLA_2_ and that the inhibitory concentration of lactadherin is lower for vesicles with a lower degree of curvature.

**Figure 4:**
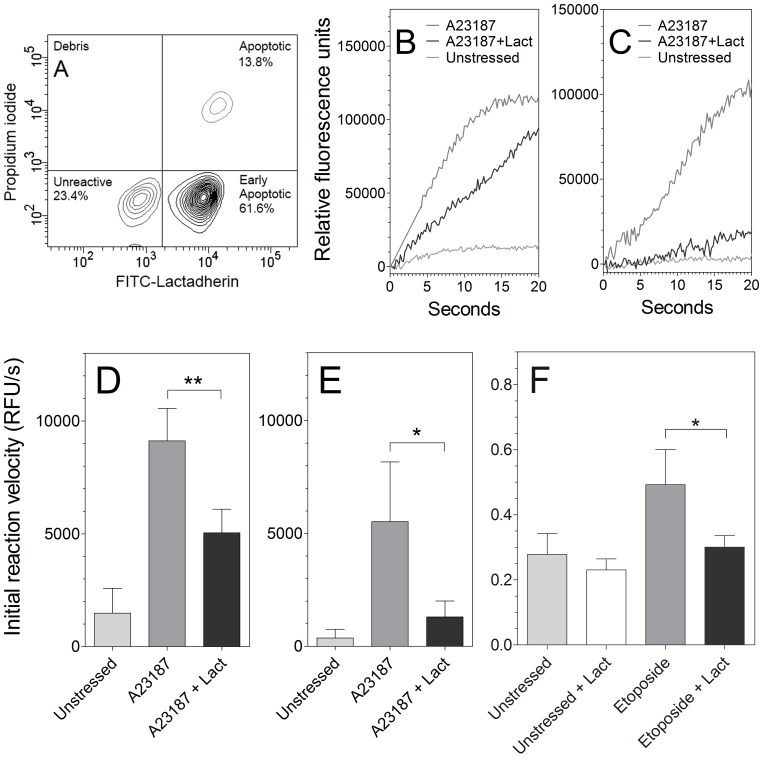
Inhibitory activity of lactadherin toward sPLA_2_’s on human leukemia cells. (**A**) NB4 cells were treated with 6 µM A23187 for 10 minutes at 22°C prior to addition of phospholipases. This treatment resulted in 61.6% pre-apoptotic cells. Phospholipase activity was detected as release of free fatty acids using the ADIFAB reagent. Each curve was run as a set in quadruplicates using a single mixture of ADIFAB and PLA_2_. Curves were run in separate sets. The initial reaction rates for nmPLA_2_ and hsPLA_2_-V were calculated by linear regression to the first 5 and 15 seconds respectively, were r^2^ = 0.85–0.95. (**B**) 0.06 U/ml nmPLA_2_ phospholipase activity on quiescent, stressed cells and stressed with 300 nM lactadherin were measured. Lactadherin was added to the cell mix immediately before adding the ionophore, proceeding with 10 minute incubation. Control curves from similar treated cells without added enzyme were subtracted as background and extrapolated Y values at injection point found using Eq. 3. Sum curves of quadruplicate sets are displayed (**C**) The experiment was repeated as in panel B using 0.06 U/ml hsPLA_2_-V. The initial reaction rate was calculated the same way as panel B. See panel **4D** and **4E** for quadruplicate results of nmPLA_2_ and hsPLA_2_-V respectively, SD displayed with *denoting p<0.05 and **denoting p<0.001. To discount any adverse interactions between A23187 and lactadherin, cells were stressed using 40 µM etoposide as described in materials and methods. As seen in Figure **4F**, inhibition of hsPLA_2_-V to near quiescent levels by addition of 300 nM lactadherin was observed, producing very similar inhibition ratios as found when using the quick, A23187 and lactadherin co-incubation protocol. Statistical significance using a one-tailed T-test assuming unequal variance showed a significance of p<0.03.

We asked whether lactadherin might also inhibit PLA_2_ activity on cell membranes (Fig. 4). For these experiments we utilized the human promyelocytic leukemia cell line, NB4. These cells were treated with 6 µM A23187 for 10 min at 22°C to stimulate pre-apoptotic PS exposure (Fig. 4A). Prothrombinase activity supported by the treated cells increased 3-fold compared to untreated cells, indicating substantial PS exposure. As judged by flow cytometry, these conditions resulted in 62% of cells meeting our criteria of substantial PS exposure without permeability to propidium iodide, while only 14% were apoptotic. To ensure that membrane structure, and consequent susceptibility to PLA_2_ were not perturbed by the assay we utilized a different assay to detect phospholipase activity. In this assay, the fluorescence of fatty acid binding protein (ADIFAB) changes on binding to free fatty acids that diffuse from the membrane after cleavage by PLA_2_. The assay was controlled by measuring the rate of free fatty acid release from cells that were quiescent, stressed, and stressed with 300 nM lactadherin in the absence of added PLA_2_. The rate of free fatty acid release was low in the untreated cells and was not significantly increased by addition of nmPLA_2_ or hsPLA_2_-V (Fig. 4B and 4C - lower curves). Addition of nmPLA_2_ or hsPLA_2_ to A23187 treated cells increased the rate of free fatty acid release by 6 and 13 fold respectively (top curves), indicating that these enzymes have little activity on quiescent cells and much greater activity on the membranes of pre-apoptotic cells. The rate of phospholipid cleavage was faster with nmPLA_2_ than with hsPLA_2_-V, but hsPLA_2_-V displayed a larger increase of the initial reaction rate on highly PS exposing cells compared to nmPLA_2_. On stressed cells we observed a decrease in fluorescence after 20–40 seconds attributable to reacylation of free fatty acids as previously described (not shown) [64], [65].

Addition of lactadherin reduced the rate of free fatty acid release (compare Fig. 4B and 4C top and middle curves). The initial rate of nmPLA_2_ activity was reduced 45% by 300 nM lactadherin on pre-apoptotic NB4 cells (Fig. 4D). Phospholipase activity of hsPLA_2_-V on these cells was reduced by >70% (Fig. 4E). To eliminate the interaction of lactadherin with A23187 as a cause of the observed inhibition, cells were stressed with 40 µM etoposide for 6 hours and run in a similar fashion. As seen in Figure 4F, 300 nM lactadherin inhibited hsPLA_2_-V to much the same extent as observed using ionophore treated cells. Adverse effects like degradation or endocytosis was discounted by doing fast runs on a Flex station 3 microplate reader using master enzyme mixes (see figure S1 and S2). The inhibitory effect of lactadherin on nmPLA2 and hsPLA2-V on plasma membranes exhibited similar characteristics as those observed using small unilamellar and large unilammellar vesicles with hsPLA2-V being more readily inhibited. This indicates the validity of using 4% PS vesicles as a reasonable pre-apoptotic plasma membrane model. Together these results indicate that secretory phospholipase A_2_ activity is increased on pre-apoptotic cells and that lactadherin inhibits the activity of the more promiscuous nmPLA_2_ as well as the more fastidious hsPLA_2_-V on pre-apoptotic human leukemia cell membranes.

## Discussion

We have shown that lactadherin inhibits activity of secretory phospholipase A_2_ on phospholipid vesicles and on cell membranes. The degree of inhibition on phospholipid vesicles is lower for the snake venom PLA_2_ than for a human secretory PLA_2_ associated with inflammation. Susceptibility to PLA_2_ inhibition by lactadherin is also influenced by membrane composition and curvature. These properties correlate to inhibition of phospholipase activity on a human leukemia cell line following cell treatment with A23187. Thus, lactadherin can inhibit secretory phospholipases A_2_ on the membranes of pre-apoptotic cells.

Our studies are in general agreement with studies indicating that some phospholipase A_2_’s can be partially inhibited by annexin A5 [66]. While the methodologies and the degree of phospholipid digestion differed substantially between the prior reports and our current report, our results are in qualitative agreement with the finding that PS-binding membrane proteins can inhibit the binding of phospholipases A_2_. It is noteworthy that the PS binding of lactadherin is calcium independent, and phosphatidyl-L-serine specific, in contrast to Annexin A5. These properties, and the incomplete overlap between binding sites for lactadherin and annexin V, likely contribute to the efficacy of lactadherin.

Our studies are in agreement with prior observations that quiescent cell membranes support little or no activity of secretory phospholipases A_2_ [17], [34], [55]. Prior studies indicated these enzymes are active on the membranes of apoptotic cells [64], [67]. Part of the explanation is likely that PS is sequestered on the inner leaflet of the resting cell membranes [29], [68]. Cellular stress induces phospholipid scrambling, which in turn exposes phosphatidylserine at the exofacial surface [34], [69]. Experiments show that PS exposing cells are target for inflammation-related secretory phospholipases like group IIA [4], [20] and V [20]. Exofacial PS exposure recruits immune cells from increased eicosanoid production via secretory phospholipase A_2_’s digestion of the plasma membrane and aids macrophage phagocytosis directly [32], [70], [71].

Secretory PLA_2_’s achieve efficacy via a 2-step mechanism [72]. First they bind to suitable sites on a membrane and subsequently cleave the sn-_2_ bonds of successive phospholipid molecules without dissociation from the membrane and re-binding [4], [73]. Formation of membrane binding sites for secretory PLA_2_ IIA and V is increased by the content of negatively charged phospholipid molecules and increased by convex curvature [65]. Binding sites for lactadherin are proportional to membrane phosphatidylserine, the major negatively charged membrane phospholipid, and increased by convex membrane curvature [41], *i.e.* formation of membrane binding sites for lactadherin is sensitive to parameters that overlap with PLA_2_’s. These similarities lead us to hypothesize that lactadherin primarily inhibits the initial binding step of secretory PLA_2_’s. As such, the results suggest that the membrane binding sites for nmPLA_2_, hsPLA_2-_V, and lactadherin overlap but are not identical.

The findings in this study identify a new potential anti-inflammatory mechanism for lactadherin. The primary, established anti-inflammatory mechanism relates to its mechanism as an opsonin for apoptotic cells. Engulfment by lactadherin-coated cells promotes an anti-inflammatory response by phagocytes [74], [75]. A second potential anti-inflammatory mechanism is the capacity to inhibit blood coagulation complexes on the membranes of cells that are stimulated, stressed, or apoptotic [45]. This study demonstrates that lactadherin may also modulate inflammation through decreasing the activity of secretory phospholipase A_2_’s. It appears possible that lactadherin may inhibit sPLA_2_’s on the same cells in which it is mediating anti-inflammatory phagocytosis during stress or apoptosis. Alternatively, secreted lactadherin may bind to cells that are remote from phagocytes and inhibit phospholipase activity independently from phagocytosis. In the present study we utilized small and large unilamellar vesicles and stressed NB4 cells to quantify the effect of lactadherin on nmPLA_2_ and hsPLA_2_-V activity. PI and FITC-lactadherin was used to monitor how many cells were in the different phases of apoptosis. PI staining (membrane permeability) in conjunction with PS exposure (stress and early apoptosis indicator) allows differentiation between stress/early apoptosis, late stage apoptosis/necrosis, cell debris and quiescent cells when combined with forward and side scatter information [76]–[78]. Stressed/early apoptotic cells stain for PS exposure, but not with PI and late stage apoptotic cells stain for PS and PI. As seen in figure 4, the primary part (∼62%) of the cell population is pre-apoptotic (PS positive, PI negative). The distinction between late stage apoptosis and necrosis can be difficult and the measured ∼14% PI/FITC-lactadherin positive cells likely indicates the death rate of NB4 cells subcultured 24 h before their use. However inhibiting phospholipases on necrotic cells enforces our conclusion, that lactadherin could function as an anti-inflammatory agent. In the presented experiments we observed efficient inhibition at 300 nM (14.1 µg/mL) lactadherin. This concentration is higher than that usually found in serum with 3–17 ng/ml for healthy adults [79], [80]. Several pathologic conditions have shown increased serum concentrations 3–40 ng/ml for childhood-onset systemic lupus patients and 13–33 ng/ml for type 2 diabetes mellitus [79], [80]. Local concentrations in the microenvironment surrounding lactadherin secreting phagocytes would however be expected to be substantially higher. Furthermore, the strong preference of sPLA2 group IIA to PS might be exploited to preferentially inhibit group IIA in rheumatoid arthritis [10]. Further studies, utilizing lactadherin fragments that mediate anti-sPLA_2_ activity independent from pro-phagocytic activity will be required to distinguish the importance of the anti-PLA_2_ activity in inflammation. These studies may indicate whether lactadherin, or a molecule with similar membrane binding properties, may have pharmaceutical value as an anti-sPLA_2_ anti-inflammatory agent.

In summary, we show that lactadherin inhibits venom (group –IA) and inflammatory (group –V) secretory phospholipase on both artificial 4% PS membranes and plasma membranes of pre-apoptotic cells. Further studies will be required to probe the inhibitory mechanism. Further studies will also be required to determine whether sPLA_2_ inhibition is a physiologic mechanism of lactadherin function or whether lactadherin, or a lactadherin-like molecule, might be adaptable for therapeutic inhibition of sPLA_2_’s.

## Supporting Information

Figure S1 **Results of nmPLA_2_ on NB4 cells as monitored by ADIFAB.** Initial experiments and optimization was done using a temperature controlled FlexStation 3 plate reader (Molecular Devices, Sunnyvale, CA). Cells were washed by gentle centrifugation at 68 RCF with slow acceleration and deceleration to avoid cell stress, followed by resuspension in 37°C no phenol red no serum RPMI 1640. 6 µM A23187 was added to the cell suspension in all samples and 300 nM lactadherin was added with A23187 in inhibition studies. 100,000 cells was dispensed per well and allowed to incubate for 10 minutes before addition of freshly made nmPLA_2_ master mix to a total concentration of 0.06 U/ml. For each 96-well plate, a column of controls were run to reference the cellular stress levels of the cells in the vial used for each run. This was done to exclude effects by different handling. Using a plate reader allowed the exclusion of any time related effects like degradation of the enzyme, ionophore, ADIFAB or similar and the total run time was 3 minutes for master mix preparation and 30 seconds until the reaction rate plateau was reached equalling less than 5 minutes total. As seen in figure S1, unstressed NB4 cells exhibited little enzymatic activity as compare to the A23187 stressed cells. Inhibiting nmPLA_2_ with lactadherin displayed similar results as observed in the spectrofluorometer with a reduction of the initial reaction rate to roughly half. In all plate reader experiments, quadruplicates where recorded and SD displayed, but the late onset of the first read (14 second delay) and the 2 second data point interval provided less than ideal data for intricate analysis, due to the partial lack of the initial reaction rate slope. A change to a high resolution spectrofluorometer system was done subsequently.
(TIFF)

Figure S2 **Results of hsPLA_2_-V on NB4 cells as monitored by ADIFAB.** Experiments using hsPLA_2_-V were carried out in an identical manner as the nmPLA_2_ experiments. Although the same 0.06 U/ml enzyme concentration was used, hsPLA_2_-V displayed less activity on stressed membranes as compared to nmPLA_2_. This behavior was later confirmed in higher resolution datasets (Fig. 4D and 4E). As seen in Figure S2, hsPLA_2_-V activity were almost completely abolished on stressed cells, however SD was larger than desired. Analyzing the data using a one-tailed T-test assuming unequal variance showed a significance of p<0.026.
(TIFF)
