# Supplementary figures and images for: Lactadherin Inhibits Secretory Phospholipase A2 Activity on Pre-Apoptotic Leukemia Cells
Source: PLoS One. 2013 Oct 23;8(10):e77143. doi: 10.1371/journal.pone.0077143 (PMC3806724; doi:10.1371/journal.pone.0077143)

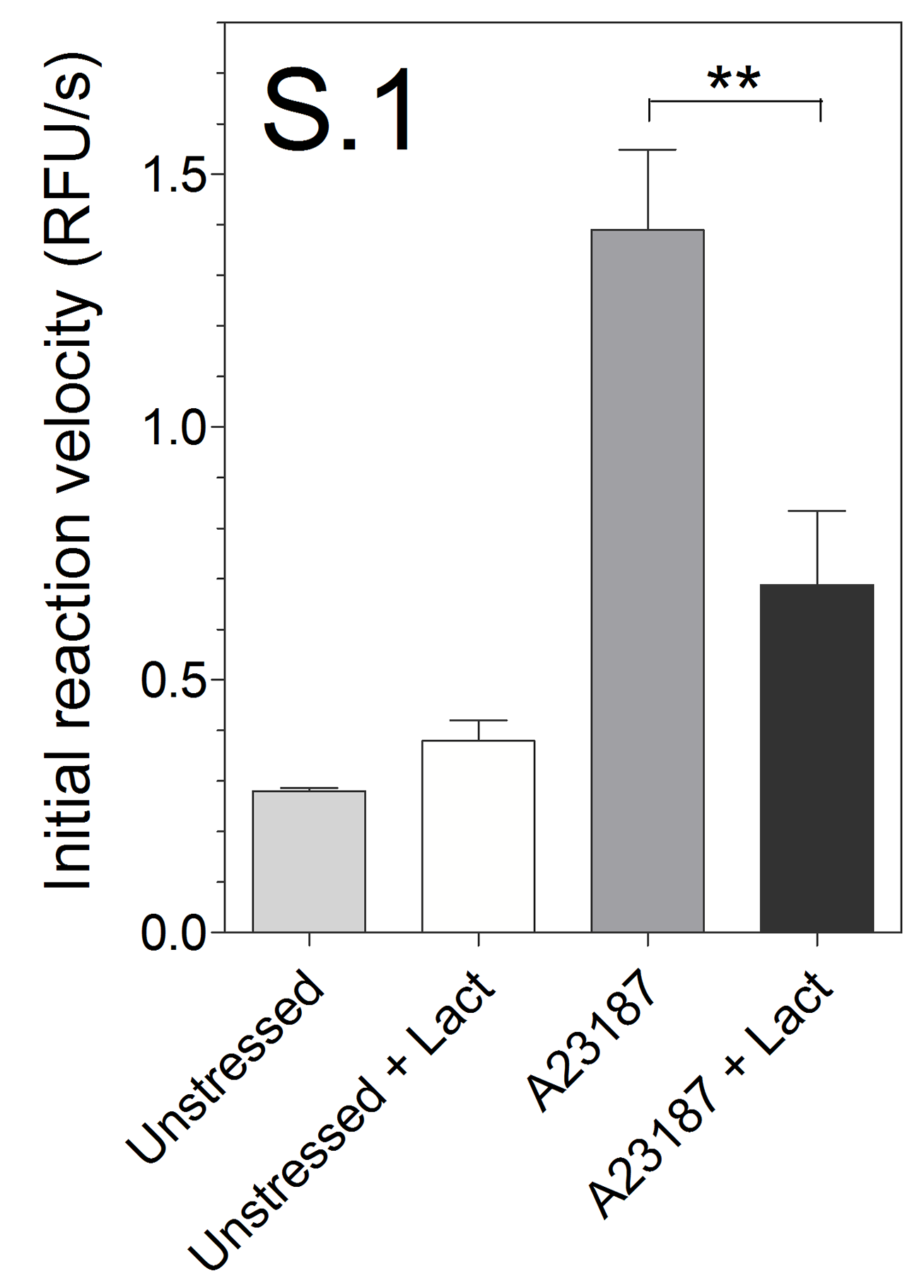

Supplement: Figure S1 — Results of nmPLA2 on NB4 cells as monitored by ADIFAB. Initial experiments and optimization was done using a temperature controlled FlexStation 3 plate reader (Molecular Devices, Sunnyvale, CA). Cells were washed by gentle centrifugation at 68 RCF with slow acceleration and deceleration to avoid cell stress, followed by resuspension in 37°C no phenol red no serum RPMI 1640. 6 µM A23187 was added to the cell suspension in all samples and 300 nM lactadherin was added with A23187 in inhibition studies. 100,000 cells was dispensed per well and allowed to incubate for 10 minutes before addition of freshly made nmPLA2 master mix to a total concentration of 0.06 U/ml. For each 96-well plate, a column of controls were run to reference the cellular stress levels of the cells in the vial used for each run. This was done to exclude effects by different handling. Using a plate reader allowed the exclusion of any time related effects like degradation of the enzyme, ionophore, ADIFAB or similar and the total run time was 3 minutes for master mix preparation and 30 seconds until the reaction rate plateau was reached equalling less than 5 minutes total. As seen in figure S1, unstressed NB4 cells exhibited little enzymatic activity as compare to the A23187 stressed cells. Inhibiting nmPLA2 with lactadherin displayed similar results as observed in the spectrofluorometer with a reduction of the initial reaction rate to roughly half. In all plate reader experiments, quadruplicates where recorded and SD displayed, but the late onset of the first read (14 second delay) and the 2 second data point interval provided less than ideal data for intricate analysis, due to the partial lack of the initial reaction rate slope. A change to a high resolution spectrofluorometer system was done subsequently. (TIFF) [file pone.0077143.s001.tiff]

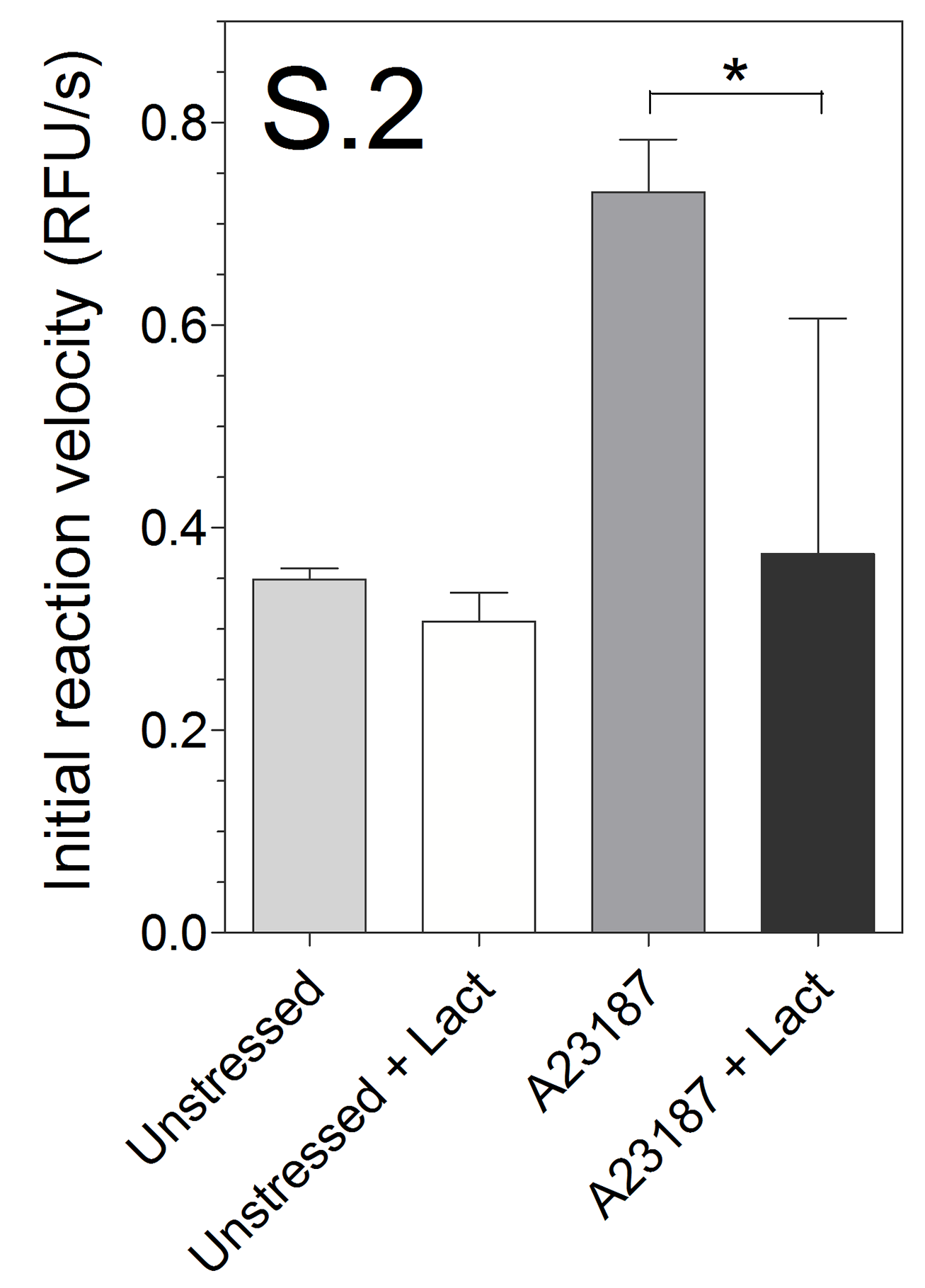

Supplement: Figure S2 — Results of hsPLA2-V on NB4 cells as monitored by ADIFAB. Experiments using hsPLA2-V were carried out in an identical manner as the nmPLA2 experiments. Although the same 0.06 U/ml enzyme concentration was used, hsPLA2-V displayed less activity on stressed membranes as compared to nmPLA2. This behavior was later confirmed in higher resolution datasets (Fig. 4D and 4E). As seen in Figure S2, hsPLA2-V activity were almost completely abolished on stressed cells, however SD was larger than desired. Analyzing the data using a one-tailed T-test assuming unequal variance showed a significance of p<0.026. (TIFF) [file pone.0077143.s002.tiff]
